# Case Report: Bacterial meningitis due to cerebrospinal fluid leakage following unilateral biportal endoscopic spinal surgery: a cautionary tale

**DOI:** 10.3389/fsurg.2024.1301905

**Published:** 2024-03-07

**Authors:** Haoyu Wang, Yunhui Wang, Zhensong Jiang, Wen Zhang

**Affiliations:** Department of Spine Surgery, Shandong Provincial Hospital Affiliated to Shandong First Medical University, Jinan, China

**Keywords:** Klebsiella pneumoniae, unilateral biportal endoscopic spinal surgery, cerebrospinal fluid, lumbar drainage, bacterial meningitis

## Abstract

Unilateral biportal endoscopic spinal surgery (UBE) is a rapidly growing surgical method and has attracted much interest recently. The most common complication of this technique is cerebrospinal fluid (CSF) leakage due to intraoperative dural tears. There have been no reports of bacterial meningitis due to dural tears in UBE surgery and its treatment and prevention. We reported a 47 year-old man with CSF due to an intraoperative dural tear. A drainage tube was routinely placed and removed on the fourth day after surgery, resulting in fever and headache on the fifith postoperative day. Blood and CSF cultures showed Klebsiella pneumoniae infection, and with lumbar drainage and appropriate antibiotics based on sensitivity tests, the patient's fever and headache were effectively relieved. This case report suggests the importance of prolonged drainage tube placement, adequate drainage, careful intraoperative separation to avoid dural tears, and effective sensitive antibiotic therapy.

## 1 Introduction

The occurrence of bacterial meningitis (BM) has been identified as a rare complication of spinal surgery, typically linked to dural rupture with cerebrospinal fluid (CSF) leakage, leading to direct bacterial invasion of the meninges (1). Incidental dural tears are relatively common during elective spinal surgery, with incidence rates ranging from 0.2% to 20%, depending on the surgical type (2). In recent years, unilateral biportal endoscopic spinal surgery (UBE), involving two independent channels for instrument operation and observation, has garnered significant interest among spine surgeons (3). The complication rate of UBE surgery ranges from 0% to 13%, with an average incidence of 6%, encompassing issues such as dural tears, recurrence, postoperative headache, incomplete decompression, nerve root injury, epidural hematoma, postoperative numbness, etc. Among these, dural tears are the most frequent, with an incidence of 2.9%–5.8% and an average of 4.1% (4). The risk of dural tears and CSF leak is up to 5.3% according to the literature (5). To our knowledge, there have been no reports of BM caused by CSF leakage after UBE surgery.

## 2 Case presentation

**Figure 1:**
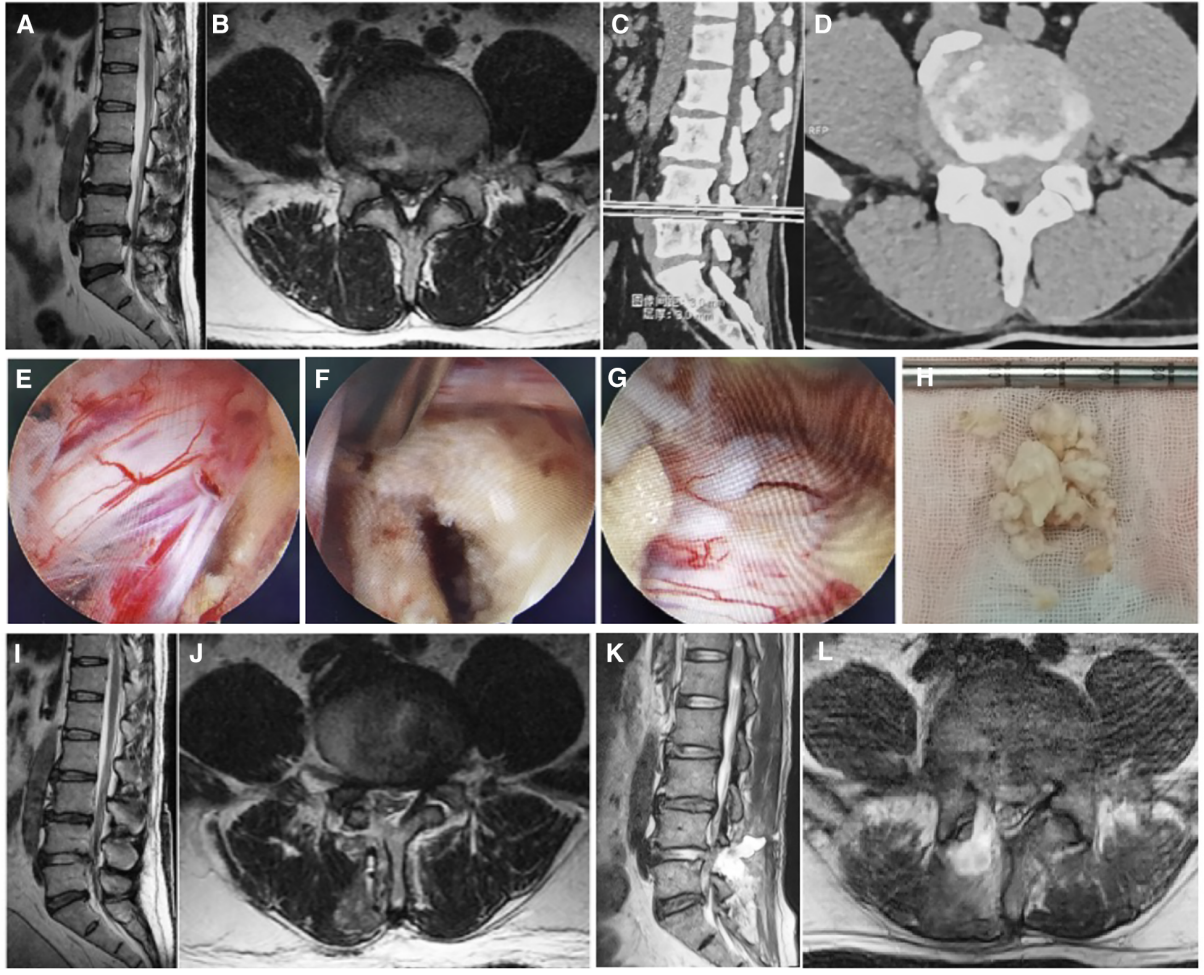
Preoperative and postoperative imaging and intraoperative pictures. Preoperative lumbar MRI images (**A,B**) and CT images (**C,D**), which show L4/5 intervertebral disc protrusion with spinal stenosis. (**E–G**) Are microscopic images during UBE surgery. (**E,F**) Show no significant compression around the nerve root after removing the nucleus pulposus; (**G**) shows a 0.3 cm tear on the dorsal side of the dura. (**H**) Represents the extracted protruding nucleus pulposus tissue. (**I,J**) Are postoperative MRI images with the protruded disc completely removed and sufficient nerve decompression. (**K,L**) Are second postoperative MRI review, indicating the accumulation of cerebrospinal fluid at the surgical site.

A 47-year-old male patient reported a history of “low back pain persisting for 10 years, intensified by radiating pain in the right lower extremity over the past 10 days.” Preoperative imaging, including MRI and CT (Figures 1A–D), revealed L4/5 intervertebral disc herniation with associated spinal stenosis. More than 10 days before admission, he experienced worsening low back pain, accompanied by radiation pain in the right lower limb. The pain extended from his right hip to the back of the right thigh, the outside of the right calf, and the dorsum of his right foot. Conservative treatment proved ineffective.

Upon admission on April 1st, the physical examination revealed the patient in a passive position, exhibiting significant limitations in lumbar flexion and extension activities. Tenderness and percussion pain were noted between the L4/5 spinous processes and paravertebral processes of the lumbar spine, radiating to the right lower limb, buttocks, outer thigh, lateral sides of the right calf, and dorsum of the foot. Shallow hypoesthesia was observed in the skin between the first toe web of the right foot. Muscle tension in both lower limbs was normal, with Grade 4 strength in the right iliopsoas muscle, right Quadriceps, right tibialis anterior muscle, and Gastrocnemius muscle. The strength of the right toe dorsal extensor muscle was Grade 3. The left iliopsoas muscle, Quadriceps, tibialis anterior muscle, and Gastrocnemius muscle strength were Grade (4+), and the left toe dorsal extensor muscle strength was also Grade (4+). The right lower limb straight leg elevation test and reinforcement test yielded positive results at 45°, while the bilateral femoral nerve traction test was negative. The left lower limb straight leg elevation test and reinforcement test were negative. Bilateral knee tendon reflexes were (+), and Achilles tendon reflexes were also (+). The patient's Babinski's sign was (−), and both ankle clonus and patellar clonus were negative. Capillaries at the ends of both lower limbs were well filled. Following admission, relevant laboratory examinations were conducted (Table 1). The patient, deemed not contraindicated for surgery, underwent unilateral biportal endoscopic (UBE) nucleus pulposus extraction under general anesthesia on January 6th. During the operation, significant compression of the right L5 nerve root was observed, along with severe adhesion between the ligamentum flavum and the dura mater. Despite meticulous separation efforts, it resulted in an approximately 0.3 cm tear in the dura mater. Following the removal of the protruding nucleus pulposus, radiofrequency hemostasis, and cleaning of the broken fibrous ring, no significant compression around the nerve root was observed, and the surgery was successfully concluded. The intraoperative image is depicted in Figures 1E–H. Postoperatively, the patient experienced significant relief from pain, and both lower limb movements were restored to their pre-surgery condition.

**Table 1:** Laboratory findings at different points in time in this case.

Date Blood analysis	1.5	1.12	1.16	1.18	1.19	1.21	1.24	1.27	1.30	2.2	2.3	2.5
WBC (10^9 ^cells/L)	9.39	9.35	6.76	8.49	7.49	9.65	8.29	7.89	6.97	5.91	5.81	5.82
RBC (10^12 ^cells/L)	4.85	4.66	4.49	4.11	4.1	4.26	4.41	4.58	4.43	4.23	4.28	4.28
HGB (g/L)	151	148	134	130	129	132	137	142	141	130	140	134
PLT (10^9^/L)	259	268	295	281	298	337	383	348	323	333	342	337
MNNO (%)	7.1	9.7	11.5	10.4	11.2	6.9	9	9.8	9.3	8.5	8.8	8.1
NEUT (%)	59.9	67.8	59.8	65.3	63.2	61.8	62.5	62.7	65.3	58	61.4	55.3
MONO (10^9 ^cells/L)	0.67	0.91	0.78	0.88	0.84	0.67	0.75	0.77	0.65	0.5	0.51	0.47
NEUT (10^9 ^cells/L)	5.62	6.34	4.04	5.54	4.73	5.96	5.18	4.95	4.55	3.43	3.57	3.22
CRP (mg/L)	1.7	25	35.7	17.7	29.7	10.6	6	3.7	3.1	1.9	1.4	1
ESR (mm/h)	6	23	42	41	42	43	28	30	20	15	14	14
Liver function												
AST (U/L, 15–40)	17	NA	NA	21	NA	NA	14	NA	NA	NA	NA	NA
ALT (U/L, 9–50)	15	NA	NA	49	NA	NA	26	NA	NA	NA	NA	NA
GGT (U/L, 11–50)	24	NA	NA	50	NA	NA	40	NA	NA	NA	NA	NA
ALP (U/L, 50–128)	54	NA	NA	66	NA	NA	65	NA	NA	NA	NA	NA
Albumin (g/L, 40–55)	61	NA	NA	33.7	NA	NA	34.6	NA	NA	NA	NA	NA
PCT (ng/ml)	NA	NA	NA	NA	0.12	NA	NA	NA	NA	0.03	NA	NA

RBC, red blood cell; WBC, white blood cell; HGB, haemoglobin; MNNO, monocyte; NEUT, Neutrophils; PLT, platelet; ESR, erythrocyte sedimentation rate; CRP, C-reactive protein; AST, aspartate aminotransferase; ALT, alanine aminotransferase; GGT glutamyl transpeptidase; ALP, alkaline phosphatase; PCT, Procalcitonin; NA, not available.

In the first, second, and third days post-surgery, approximately 200 ml, 250 ml, and 200 ml of clear cerebrospinal fluid (CSF) were drained through the patient's incision drainage tube. The heightened drainage was attributed to CSF leakage, prompting appropriate fluid replacement to prevent electrolyte disorders. Concurrently, a postoperative review of the lumbar MRI revealed complete removal of the protruded disc and adequate nerve decompression, as illustrated in Figures 1I,J.

**Figure 2:**
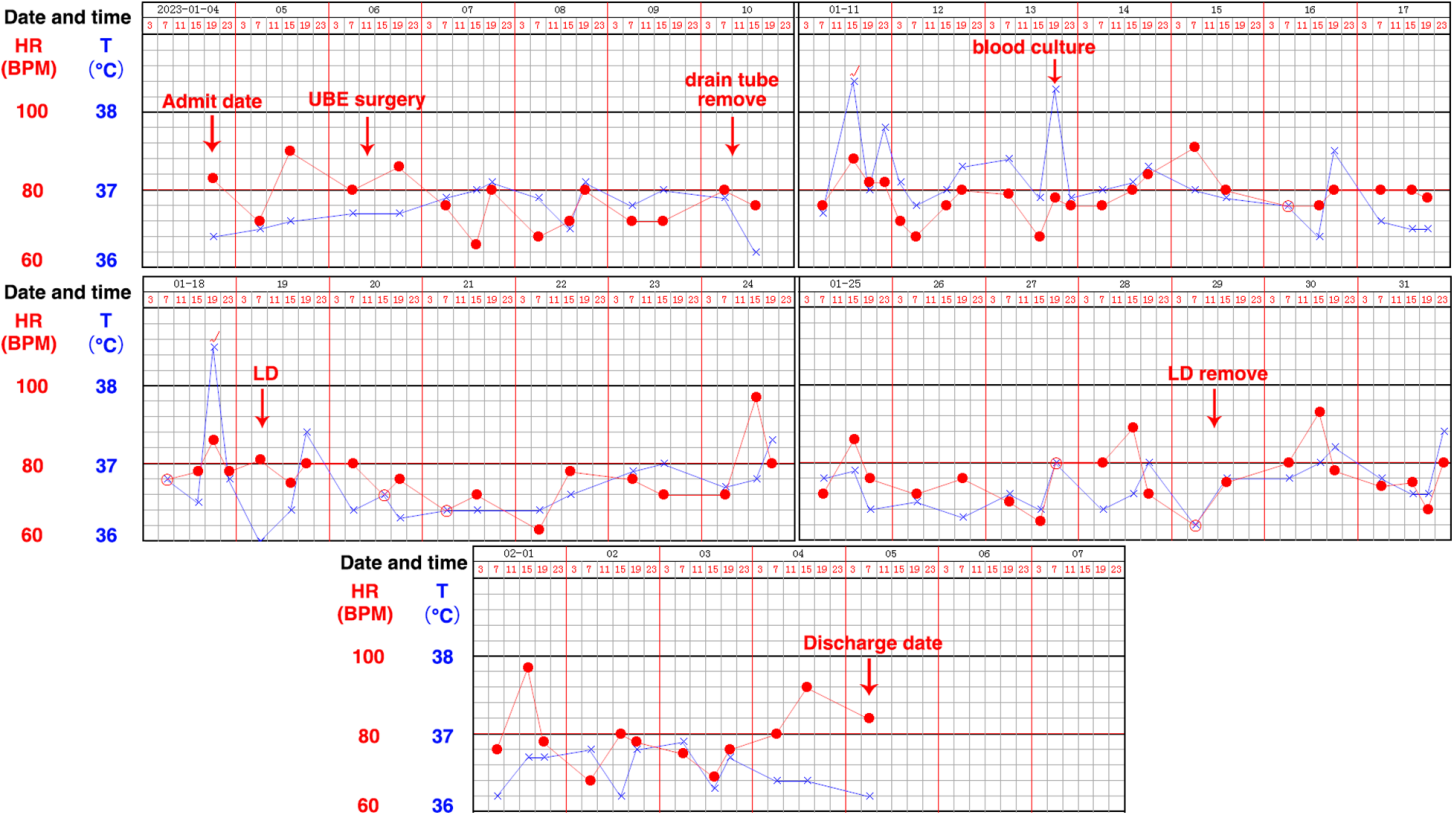
The patient's temperature record sheet. LD: Lumbar drain (LD) placement is a common neurosurgical procedure in which a fine spinal catheter is temporarily inserted into the lumbar spinal canal and connected to a closed system for controlled diversion of CSF. The figure clearly records the changes in the patient's body temperature at various times, including the temperature at admission, the temperature after UBE surgery, the temperature after removing the drainage tube, the temperature during blood culture, the temperature of the LD, and.

On the 4th day post-surgery (January 10, the fourth day after operation), 100 ml of clear cerebrospinal fluid (CSF) was drained, and subsequently, the drainage tube was removed. At 15:00 on the afternoon of the following day (January 11, the fifth day after surgery), after the removal of the drainage tube, the patient developed headaches and fever (Figure 2). It was presumed that the patient, experiencing CSF leakage, might exhibit a fever reaction due to localized residual CSF stimulation post-drainage tube removal. However, at 19:00 on the third day (January 13, the seventh day after surgery) after the removal of the drainage tube, the patient once again presented with a high fever and a headache. Suspecting a potential postoperative infection, blood culture was conducted for the first time, and the patient's vital signs and electrolyte balance were closely monitored. The patient's fever and headache symptoms persisted for four days without noticeable relief. During this period, blood culture results revealed Klebsiella pneumoniae, and meropenem was administered based on drug sensitivity testing (1 g, three times a day; administered from January 17 to January 18). At 19:00 on the 7th day post-drainage tube removal (January 18, the twelfth day after surgery), the patient once again experienced a high fever (38.5°C) accompanied by a headache. The following day (January 19, the 13th day after surgery), a routine blood examination indicated elevated levels of CRP and procalcitonin (Table 1). During this period, a re-examination of the lumbar MRI (Figures 1K–L) revealed CSF accumulation at the operation site, with no apparent abnormalities found in the brain MRI. Initially, the plan was to guide the placement of a drainage tube under ultrasound, but ultrasound indicated a small capsule cavity with fluid pulsation in the capsule (Supplementary Videos 1 and 2), resulting in an unsuccessful tube placement. Subsequently, the decision was made to perform the first lumbar puncture, revealing a significant increase in intracranial pressure and turbid CSF. Considering the possibility of bacterial meningitis (BM), it was decided to insert a lumbar drain (LD). Following the drainage, the patient's symptoms were alleviated, and the temperature decreased (Figure 2). CSF analysis indicated glucose levels of 0.31 mmol/L (normal range: 3.9–6.1), protein levels of 1.94 g/L (normal range: 0.15–0.45), chloride levels of 121.8 mmol/L (normal range: 99–110), nucleated cells at 2,807 × 10^6^/L, and the CSF appeared colorless and turbid, with a positive Pandy's test (Table 2). On the day of lumbar drain placement (January 19, the 13th day after surgery), CSF bacterial culture results identified Klebsiella pneumoniae, confirming the diagnosis of bacterial meningitis (BM). Following consultation with the clinical pharmacy department, meropenem was discontinued, and imipenem cilastatin was initiated (1 g, three times a day, administered from January 19 to February 3). The lumbar drain (LD) tube was safely placed, and CSF drainage was controlled at approximately 150–200 ml/day. The CSF bacterial culture results remained positive for Klebsiella pneumoniae on the second day (January 20, the 14th day after surgery) after LD tube placement. However, with the combined effect of the lumbar drain and appropriate antibiotics based on sensitivity testing, white blood cell counts and CRP levels normalized by the sixth day (January 24, the 18th day after surgery), and the CSF bacterial culture on Day 1.26 turned negative. The patient's routine blood inflammatory indicators continued to decrease, and glucose and protein levels in the CSF gradually returned to normal. The patient's fever and headache symptoms were completely relieved. The results of various routine blood tests, including calcitonin, CRP, ESR, and other laboratory tests, are detailed in Tables 1, 2. On January 29 (The 23rd day after surgery), the lumbar drain (LD) tube was removed, and after two consecutive normal CRP readings, the patient's treatment was transitioned to oral antibiotic levofloxacin tablets (0.5 g once a day). Subsequent reexaminations of WBC, CRP, and ESR all showed normal levels, leading to the patient's discharge from the hospital. As of the latest follow-up, the patient has not reported any discomfort within six months post-surgery.

**Table 2:** CSF analysis and bacterial culture at different points in time in this case.

Date CSF analysis and bacterial culture	1.13	1.16	1.18	1.19	1.20	1.22	1.26
CSF analysis							
RBC count (10^6^/L)				Colourless	2,000	1,000	1,000
Nucleated cell count (10^6^/L)				2,807	1,814	1,409	283
Monocytes count (10^6^/L)				1,146	1,265	1,011	239
Monocytes proportion (%)				40.8	69.7	71.7	84.4
Multinucleated cell count (10^6^/L)				1,661	549	398	44
Multinucleated cell proportion (%)				59.2	30.3	28.3	15.6
Pandy's test				2+	2+	1+	1+
GLU (mmol/L)				0.31	NA	NA	2.39
Cl (mmol/L)				121.8	NA	NA	119.8
ADA (U/L)				6.3	NA	NA	2.8
PR (g/L)				1.94	NA	NA	1.08
Bacterial culture							
Blood culture	+	−	−	NA	NA		NA
CSF culture	NA	NA	NA	+	+	−	−

CSF, cerebrospinal fluid; RBC, red blood cell; GLU, glucose; Cl, chloride; ADA, adenosine deaminase; PR, Protein quantitative; (+), positive; NA, not available, negative; (+), positive.

## 3 Discussion

CSF leakage, occurring in 0.2% to 20% of cases depending on the type of surgery (2), is a not uncommon intraoperative complication associated with spinal surgery. It can result in intracranial hypotension, surgical site infection, pyogenic meningitis, and prolonged hospitalization (6). Postoperative bacterial meningitis (BM) is a rare and severe complication linked to spinal surgery, often stemming from intraoperative dural tears with CSF leakage, leading to direct bacterial invasion of the meninges (1). Common organisms reported in BM include Staphylococcus aureus, Escherichia coli, and Enterococcus faecalis (7). Given the low incidence of BM after spinal surgery, literature on its development and treatment is limited. This report represents the first documentation of BM due to dural tears in unilateral biportal endoscopic (UBE) surgery, detailing its treatment and prevention.

The unilateral biportal endoscopic (UBE) technique is a rapidly advancing surgical approach utilizing an arthroscopic system for treating spine diseases. UBE offers numerous advantages, including enhanced surgical instrument movement with independent visualization and working portals, reduced bleeding due to continuous irrigation, expansive and clear visualization for unhindered access to contralateral and foraminal areas, use of standard arthroscopy and spinal instruments, and improved surgical manipulation for effective decompression (8). However, like any new technology, UBE is not without unexpected complications. The complication rate ranges from 0% to 13%, averaging 6%, encompassing issues such as dural tears, recurrence, postoperative headache, incomplete decompression, nerve root injury, epidural hematoma, and postoperative numbness. Dural tears are the most prevalent, with an incidence ranging from 2.9% to 5.8% and an average of 4.1% (4). Dural tears during unilateral biportal endoscopic (UBE) surgery primarily result from a lack of familiarity with the technique in the early stages of adoption, coupled with the potential for inadvertent injury to the dura during the manipulation of the yellow ligament and removal of the ligamentum flavum. To prevent such injuries, it is crucial for the surgeon to exercise care when separating the dura from the ligamentum flavum while removing it along the path of the nerve root (9).

During the operation on this patient, a dorsal dural tear occurred while removing the ligamentum flavum. No suturing was performed due to the occlusion of the spinous process. A drain tube was placed at the surgical site and removed on the 4th postoperative day, still yielding 100 ml of drainage fluid. Following drainage tube removal, the patient developed pyrexia (38.4 °C) and severe headaches, with blood culture results revealing Klebsiella pneumoniae infection. Despite timely antibiotic administration, the patient's pyrexia and headache symptoms did not significantly improve. Lumbar spine MRI indicated a focal fluid collection within the paraspinal musculature and surgical bed at the level of L4/5, suggesting a potential surgical site infection. The infected cerebrospinal fluid (CSF) entered the subdural space through dural rupture, leading to bacterial meningitis (BM).

When the initial attempt using ultrasound guidance to insert a drainage tube failed due to the small cyst cavity, we opted for lumbar drain (LD) placement. LD placement is a common neurosurgical procedure involving the temporary insertion of a fine spinal catheter into the lumbar spinal canal, connected to a closed system for controlled diversion of cerebrospinal fluid (CSF), serving both diagnostic and therapeutic purposes (10). Under local infiltration anesthesia, a lumbar puncture needle was positioned in the L3/4 interspace. Upon CSF outflow, the drainage catheter was implanted into the subdural space, and the valve was promptly closed to prevent further CSF release. Subsequently, the drainage catheter was securely fixed onto the patient's back. The LD tube was placed at a safe height, with CSF drainage controlled at approximately 150–200 ml/day. In cases where the patient experienced a severe headache due to intracranial hypotension, the LD catheter could be temporarily closed or raised to decrease CSF drainage (11).

The cerebrospinal fluid (CSF) analysis revealed low sugar, high protein, and an increased number of nucleated cells, indicative of bacterial meningitis (BM). CSF culture results aligned with the blood culture, confirming Klebsiella pneumoniae infection. Considering the patient's clinical symptoms, CSF analysis, and culture results, the diagnosis of BM was evident. Following sufficient lumbar drain (LD) management and timely adjustment of antibiotics based on drug sensitivity testing, the patient experienced notable relief from headache and fever symptoms.

In this case, despite the use of effective antibiotics following the blood culture results, the patient's headache and fever symptoms were not adequately relieved. This may be attributed to the low effective concentration of antibiotics in the cerebrospinal fluid (CSF) and the delayed clearance of bacterial-contaminated CSF (1). It could also explain why symptoms were not significantly alleviated before lumbar drain (LD) placement with the same antibiotics, while significant relief was observed after the LD tube was inserted. Upon reviewing the entire treatment process, there's a possibility that the drainage tube might have been removed too early post-surgery. In future cases, consideration should be given to delaying extubation and removing the drainage tube when the drainage fluid is significantly reduced. This approach may help avoid the need for LD and contribute to symptom relief.

The history and treatment process of the case we shared were relatively simple, but they have a good warning effect. First, this case reminded us that whether in the process of open surgery or minimally invasive surgery, we should carefully separate the adhesions and operate carefully to try to avoid CSF leakage due to dural tears. Second, if CSF leakage occurs after surgery, the drainage time should be appropriately extended, and sufficient drainage should be carried out to avoid the formation of dead space, which can facilitate bacterial infections (12). Third, when removing the drainage tube of patients with CSF leakage after surgery, we should routinely send the drainage fluid for analysis and culture to provide us with the treatment direction when there is a bacterial infection. Finally, lumbar drainage can be a complementary means of adequate drainage if BM is considered in patients with CSF leakage after postoperative removal of the drainage tube, which is why we share this case. To our knowledge, this study is the first to report BM caused by CSF leakage after UBE surgery. After six months of follow-up, the patient's symptoms in the right lower limb were completely relieved, no fever or headache symptoms appeared, and satisfactory results were achieved.

In conclusion, we have presented a case of bacterial meningitis (BM) caused by dural tears in unilateral biportal endoscopic (UBE) surgery, detailing its treatment and prevention. The case involved a 47-year-old man with cerebrospinal fluid (CSF) leakage due to intraoperative dural tear. Routine placement and removal of a drainage tube led to fever and headache, with subsequent blood and CSF cultures confirming Klebsiella pneumoniae infection. Lumbar drain (LD) placement and appropriate antibiotic therapy, guided by sensitivity testing, effectively relieved the patient's fever and headache. This case underscores the significance of prolonged drainage tube placement, ensuring adequate drainage, careful intraoperative maneuvers to prevent dural tears, and the implementation of effective antibiotic therapy based on sensitivity testing.

## Data Availability

The original contributions presented in the study are included in the article/Supplementary Material, further inquiries can be directed to the corresponding authors.
